# Comparison of Four Bisulfite Conversion Kits and Their Effects on DNA Methylation Detection and Age Prediction

**DOI:** 10.3390/genes17091100

**Published:** 2026-09-11

**Authors:** Monica Giuffrida, Claus Børsting, Vania Pereira

**Affiliations:** Section of Forensic Genetics, Department of Forensic Medicine, Faculty of Health Sciences, University of Copenhagen, 11 Frederik V’s Vej, DK-2100 Copenhagen, Denmark; monicagiuffrida@sund.ku.dk (M.G.); vania.pereira@sund.ku.dk (V.P.)

**Keywords:** bisulfite treatment, massively parallel sequencing, low-input DNA, epigenetics, forensic genetics, CpG

## Abstract

**Background/Objectives**: This study aimed to compare the performance of four commercial bisulfite conversion kits—the Premium Bisulfite Kit, the SuperMethyl™ Fast Bisulfite Conversion Kit, the SuperMethyl™ Max Bisulfite Conversion Kit, and the EZ DNA Methylation-Gold™ Kit (hereafter: Premium, Fast, Max, and Gold)—to evaluate their impact on the age prediction accuracy at different DNA input levels. **Methods**: Comparisons were performed using DNA extracted from blood samples and DNA methylation standards. Different inputs of DNA were tested (1–50 ng), and all samples were analyzed twice. After bisulfite treatment, 10 targets were amplified by PCR and sequenced on a MiSeq instrument. Performance was evaluated across several metrics: DNA yield after PCR, conversion efficiency, read depth and β-value reproducibility, β-value specificity, cost, and hands-on time. Furthermore, DNA methylation levels of age-associated CpGs on the 10 PCR fragments were investigated, and the accuracy of age predictions was determined. **Results**: No kit was superior in performance across all metrics. Higher post-PCR DNA concentrations and more robust and reproducible results, particularly at lower DNA inputs, were obtained after treatment with the Premium and Max kits. The amplicon concentrations, read depths, and reproducibility between duplicate determinations were significantly lower with 1 ng DNA input compared to inputs of 5–50 ng DNA. **Conclusions**: For forensic genetic age prediction purposes, the Max or Premium kits represented more suitable options for bisulfite conversion, whereas the Fast kit may be preferable in settings where sample quantity is not a limiting factor, and cost or throughput are the primary considerations.

## 1. Introduction

DNA methylation primarily occurs at the fifth carbon of cytosines (5 mC) followed by a guanine residue (G) [1,2]. These genomic locations are known as CpG sites [3] and play crucial roles in regulating gene expression, organizing chromatin structure, and cell differentiation [4]. In the field of forensic genetics, DNA methylation is applied for tissue identification, distinguishing between monozygotic twins, and age estimation [5,6,7,8,9].

Over the last two decades, sequencing of bisulfite-treated DNA has established itself as the leading approach for DNA methylation analysis [10,11]. The method involves bisulfite treatment of DNA, which translates the methylation status of CpG sites—an otherwise undetectable epigenetic modification—into genomic information that may be analyzed by massively parallel sequencing (MPS). The first bisulfite conversion protocol was developed in 1992 [12]. The treatment causes deamination of unmethylated cytosines into uracils (U), while leaving the methylated cytosines unmodified. The bisulfite-treated DNA is subsequently amplified by PCR, where the DNA polymerase converts the uracils (U) into thymines (T). The DNA methylation level at a CpG site can be determined by the proportion of amplified DNA molecules with either cytosines (C) or thymines (T). Among the many available methodologies, MPS is the most effective for quantifying C/T levels at CpG sites, and hence estimating DNA methylation proportions, in terms of accuracy, efficiency, resolution, and sensitivity [8,11,13,14].

Bisulfite conversion causes DNA loss and fragmentation due to the high temperatures and acidic pH required for the reaction to occur [15,16]. The DNA damage may be reduced by lowering the incubation temperatures, bisulfite concentration, or the DNA exposure time to bisulfite, but the downside may be incomplete bisulfite conversion and inaccurate estimations of the methylation levels [16]. To ensure optimal conversion and high DNA recovery rates, standard bisulfite conversion protocols require up to 16 h and large DNA input amounts per reaction (50–500 ng) [12].

The amount of DNA obtained from crime scene samples is often limited and, therefore, incompatible with the requirements of the standard bisulfite conversion methods. Previous studies compared the performance of several commercially available bisulfite conversion kits [17,18,19,20,21,22,23]. The studies confirmed that only a small amount of DNA is recovered after the bisulfite treatment, especially when the DNA inputs were low (<10 ng) [20,21,24]. However, two cutting-edge bisulfite conversion kits were recently released: the SuperMethyl™ Fast Bisulfite Conversion kit (Ellis Bio, Cambridge, MA, USA) (hereafter: Fast kit) and the SuperMethyl™ Max Bisulfite Conversion kit (Ellis Bio, Cambridge, MA, USA) (hereafter: Max kit). The Fast kit incorporates an optimized ultrafast bisulfite technology [25], whilst the Max kit uses an ultra-mild bisulfite treatment [26] due to either a short exposure to sodium bisulfite or a different composition of the bisulfite solution in the conversion step. High conversion efficiencies were reported for DNA inputs as low as 10 ng for the Fast kit, and 100 pg for the Max kit [27].

The aim of this study was to evaluate and test the performance of two established bisulfite conversion kits [9,17,18,19], the EZ DNA Methylation—Gold™ kit (Zymo Research, Irvine, CA, USA) (hereafter: Gold kit) and the Premium Bisulfite kit (Diagenode, Liege, Belgium) (hereafter: Premium kit), against the two novel bisulfite kits. The four bisulfite conversion kits (Premium, Gold, Fast, and Max) differ in duration and temperatures of the bisulfite-incubation step, hands-on time, methodology employed in the purification steps (bead- or column-based), and elution volumes (Table 1). The methylation levels obtained with the Premium, Gold, Fast, and Max kits were compared in their performance, reproducibility, and sensitivity across different DNA input amounts. Furthermore, age predictions were carried out using two previously developed models currently used in the forensic field, the 11-CpG model [9] and the SVMp-34CpG model [28], and the performance of the four kits was compared.

## 2. Materials and Methods

### 2.1. Samples and DNA Methylation Standards

A total of 10 blood samples from five male and five female adult individuals [9] and five DNA methylation standards (percentage of methylation: 0%, 25%, 50%, 75%, and 100%) were analyzed. The blood samples were obtained from fully anonymized blood donors in the Capital Region of Denmark, whose broad consent to participate in research studies was obtained upon routine blood donation. Since the samples were fully anonymized and this study does not qualify as health-related research, an ethical approval was not required, in accordance with Danish ethical guidelines and legislation. The age span of the blood donors at the donation points ranged from 18 to 68 years. The five DNA methylation standards were created by combining the Human Methylated & Non-methylated (WGA) DNA Set (Zymo Research, Irvine, CA, USA) in varying proportions. An overview of the blood samples and DNA methylation standards used in this study is shown in Supplementary Table S1.

### 2.2. Sample Preparation and Quantification

The DNA from the blood samples had previously been extracted with the QIAamp DNA Mini Kit (Qiagen, Hilden, Germany) and stored at −20 °C. Blood samples and DNA methylation standards were quantified with the Qubit dsDNA High Sensitivity (HS) Assay Kit (Thermo Fisher, Waltham, MA, USA) using a QuBit 2.0 Fluorometer (Thermo Fisher Scientific) and diluted to a concentration of 2.5 ng/µL (hereafter: 50 ng cohort). Three of the blood samples (18, 42, and 68 years old) were further diluted to 0.5 ng/µL, 0.25 ng/µL, and 0.05 ng/µL (hereafter: sensitivity cohort).

### 2.3. Bisulfite Conversion

All blood samples and DNA methylation standards with DNA input ranging from 50 to 1 ng (2.5–0.05 ng/µL) underwent bisulfite conversion with the four commercial kits: the Premium Bisulfite Kit, the SuperMethyl™ Fast Bisulfite Conversion Kit, the SuperMethyl™ Max Bisulfite Conversion Kit, and the EZ DNA Methylation-Gold™ Kit. For each kit, samples were processed in duplicates according to the manufacturers’ protocols. A negative control was included in each run. Differences among the four laboratory protocols are summarized in Table 1.

### 2.4. Amplification, Library Preparation and Sequencing

The PCR amplification, library preparation, pooling, and sequencing of the samples and DNA methylation standards were performed as previously described [9]. Ten fragments were amplified in two multiplex PCR reactions with 4 and 6 targets, a 4-plex and a 6-plex, respectively, containing relevant CpGs previously used for age prediction [9,28,29]. Eight sequencing runs were performed, two per bisulfite conversion kit.

### 2.5. Statistical and Data Analysis

Base call files from the sequencing instrument were processed as previously described [9,28]. Information about locus read depth, bisulfite conversion efficiency, methylation values (β-values), and the age predictions using the 11-CpG [9] and the SVMp-34CpG [28] models were generated. The downstream data analyses were performed in R v4.4.1 [30]. The performance of the four kits was evaluated by comparing: (i) DNA concentrations after bisulfite conversion and PCR, (ii) bisulfite conversion efficiency, (iii) read depth and β-value reproducibility among duplicate runs, (iv) β-values specificity in DNA methylation standards and in the sensitivity cohort, (v) age predictions. Point (i) aimed to investigate the DNA concentrations after bisulfite treatment and PCR amplification in the samples with 50 ng input and in the sensitivity cohorts to evaluate which kit best preserved the genetic material. The analysis in point (ii) focused on bisulfite conversion efficiencies of the kits across different DNA inputs (50 to 1 ng). Point (iii) compared the read depth and β-value reproducibility across duplicate runs of samples processed with each kit. The β-values were also investigated in point (iv), with a focus on the specificity in the DNA methylation standards and at lower DNA inputs (closeness of the observed β-values to their expected values). Point (v) compared the age prediction errors in the samples with 50 ng input and the sensitivity cohorts across the four tested bisulfite conversion kits. An overview of the statistical tests performed in this study can be found in Supplementary Table S2.

#### 2.5.1. Multiplex Quantification

The PCR products from the two multiplexes were quantified with the Qubit dsDNA High Sensitivity (HS) Assay Kit and the QuBit 2.0 Fluorometer. Results were compared using an in-house script with the aid of the packages: readxl v1.4.5 [31], tidyverse v2.0.0 [32], lme4 v1.1.37 [33], lmerTest v3.2.1 [34], emmeans v2.0.2 [35], and dunn.test v1.3.7 [36]. A linear mixed model per multiplex (4-plex and 6-plex) was fitted with kit as fixed effect and sample as random effect to account for repeated measures across kit and sequencing runs, using the package lme4 v1.1.37. Model assumptions were assessed via residual diagnostics. The package lmerTest v3.2.1 was employed to perform a Type III ANOVA on the linear mixed model output to evaluate whether there was a significant difference in the DNA concentrations. Benjamini-Hochberg (BH-adjusted) post-hoc pairwise comparisons were performed using the package emmeans v2.0.2 to assess pairwise differences in concentrations obtained using different bisulfite kits.

DNA concentrations obtained from the sensitivity cohort were compared to the results from the negative control that was used as background noise reference. To assess whether the sensitivity cohort concentrations showed a signal significantly above the negative control background noise, one-sample Wilcoxon signed-rank tests against zero were performed using base R on the signals’ differences. Furthermore, non-parametric Kruskal-Wallis tests were performed using base R, followed by Dunn post-hoc tests with Benjamini-Hochberg correction using dunn.test v1.3.7, to compare signal differences among different bisulfite kits.

#### 2.5.2. Bisulfite Conversion Efficiency

Read counts of the cytosines and thymines at non-methylated cytosine positions distributed across the 10 amplicons were obtained as previously described [9]. For each of these genomic positions, the bisulfite conversion efficiency was calculated as T/C+T and the resulting values averaged per sample or DNA methylation standard. Bisulfite conversion efficiencies were investigated across kits using an in-house script employing the tidyverse v2.0.0, lme4 v1.1.37, lmerTest v3.2.1, emmeans v2.0.2, and dunn.test v1.3.7 packages.

First, Kruskal-Wallis and Dunn post-hoc comparison tests were performed for each bisulfite kit using base R and the package dunn.test 1.3.7, respectively, to evaluate possible differences in conversion efficiency across input levels (50 ng, 10 ng, 5 ng, and 1 ng). Next, the bisulfite kits were compared to each other using a linear mixed model (package lme4 v1.1.37), fitted using kit as fixed effect and sample identity as random effect, to account for replicates of the same samples across kits and runs. Model assumptions were assessed via residual diagnostics. A Type III ANOVA (package lmerTest v3.2.1) was performed on its output. BH-adjusted post-hoc comparisons (package emmeans v2.0.2) were assessed in pairs of bisulfite kits to identify the kit that presented the highest bisulfite conversion efficiency.

#### 2.5.3. Read Depth and β-Value Reproducibility

Read depth reproducibility was investigated using two complementary metrics: total read depth and normalized read depth. The analyzed CpGs were those of the original selection [29], although only one of the CpGs in the ELOVL2 amplicon (cg24724428) was considered for the read depth data analyses. Total read depth was calculated as the sum of all the C and T reads at the CpG sites, while the normalized read depth was calculated as the number of reads (C + T) of each amplicon divided by the total read depth. An in-house script with the aid of the tidyverse v2.0.0, lme4 v1.1.37, lmerTest v3.2.1, emmeans v2.0.2, and dunn.test v1.3.7 packages was employed for the read depth analyses. Read depth reproducibility was investigated both in the 50 ng cohort and across different DNA inputs (10 ng, 5 ng, and 1 ng) using the sensitivity cohort. A linear mixed model (package lme4 v1.1.37) with kit as fixed effect and sample as random effect to account for sample replicates, a Type III ANOVA (package lmerTest v3.2.1), and BH-adjusted pairwise comparisons (package emmeans v2.0.2) were used to investigate significant differences among kits in total and normal read depth. Model assumptions were assessed via residual diagnostics. Kruskal-Wallis tests and BH-adjusted Dunn post-hoc comparisons (base R and package emmeans v2.0.2) were performed at the different DNA input levels to reveal any significant differences among kits in total and normalized read depth. Additionally, the sensitivity cohort and their reference samples from the 50 ng cohort were compared using a linear mixed model, Type III ANOVA, and BH-adjusted comparisons to assess at which DNA input the total read depth became significantly lower.

The β-value reproducibility, reflecting whether the methylation values themselves were consistent between sequencing runs, was investigated among bisulfite conversion kits using another in-house script and the packages readxl v1.4.5, tidyverse v2.0.0, lme4 v1.1.37, lmerTest v3.2.1, and emmeans v2.0.2. The mean absolute difference (MAD) between the β-values of the 11 CpGs [29] obtained from the two runs for each sample was calculated. A Type III ANOVA (package lmerTest v3.2.1) was performed on the linear mixed model (package lme4 v1.1.37) fitted on the MADs, with kit as fixed effect and sample and DNA input as random effects, to account for different DNA inputs and sample replicates. Model assumptions were checked via residual diagnostics. BH-adjusted pairwise comparisons (package emmeans v2.0.2) were used to assess whether any kit presented significant better β-value reproducibility across sequencing runs.

#### 2.5.4. β-Value Specificity in DNA Methylation Standards and Sensitivity Cohort

The specificity of the β-values of the DNA methylation standards was investigated across the 11 CpGs [29] using an in-house script and the tidyverse v2.0.0, lme4 v1.1.37, lmerTest v3.2.1, and emmeans v2.0.2 packages. The β-values across the 11 CpGs were calculated as C/C+T, averaged per DNA methylation standard, and converted to percentages for direct comparison with the expected methylation percentages. The absolute difference and bias were calculated for each DNA methylation standard using the mean observed methylation percentage and the expected value. A linear mixed model (lme4 v1.1.37) was created with kit as fixed effect, expected methylation level as covariate, and sequencing replicate nested within kit as a random effect to account for the duplicate runs of the same DNA methylation standards. Model assumptions were checked via residual diagnostics. A Type III ANOVA (package lmerTest v3.2.1) was performed on the model output to assess the significant effect of either kit or methylation level on the β-value specificity. BH-adjusted post-hoc comparisons (package emmeans v2.0.2) were further performed to assess significant differences in β-value specificity between kits.

Likewise, the β-values obtained at the 11 CpG sites in the sensitivity cohort were further investigated with an in-house script using the packages readxl v1.4.5 and tidyverse v2.0.0. The β-values of the sensitivity cohort were compared to the β-values of the corresponding samples in the 50 ng cohort. For each CpG, β-values were averaged across the two duplicate runs at each input level and in the 50 ng reference. Non-parametric Kruskal-Wallis tests were performed using base R to assess whether the choice of bisulfite kit affected the β-value specificity and with which kit the samples presented the most accurate estimates of the β-values at lower DNA input.

#### 2.5.5. Age Prediction

Age predictions were generated using the 11-CpG model [9] and the SVMp-34CpG model [28] and compared across bisulfite conversion kits and DNA inputs. Age prediction performance was investigated with an in-house script using the packages tidyverse v2.0.0, lme4 v1.1.37, lmerTest v3.2.1, emmeans v2.0.2, dunn.test v1.3.7, and readxl v1.4.5. Predictions from duplicate sequencing runs were averaged prior to error calculation. The 50 ng cohort samples were investigated with Type III ANOVAs (package lmerTest v3.2.1) on the linear mixed models (lme4 v1.1.37) fitted on the prediction errors of each model, using kit as fixed effect and sample as random effect. The statistical tests accounted for repetitive samples and compared prediction errors to assess the effect of the bisulfite kit on prediction accuracy. Model assumptions were checked via residual diagnostics. At lower DNA inputs (1, 5, and 10 ng), where sample sizes were limited (3 samples per kit), non-parametric Kruskal-Wallis tests were used instead.

Additionally, to compare the two prediction models, an additional linear mixed model was fitted with model type and DNA input as fixed effects and sample identity and kit as random effects. Model assumptions were checked via residual diagnostics. The significance of the model effect, the DNA input effect, and their interaction were assessed using Type III ANOVAs and BH-adjusted pairwise comparisons (package emmeans v2.0.2). The interaction between model type and DNA input was tested to assess whether the relative performance of the two models was consistent across input levels. Finally, to identify the DNA input threshold at which prediction accuracy significantly deteriorated, BH-adjusted pairwise comparisons between input levels were extracted from the interaction model.

## 3. Results

### 3.1. Multiplex Quantification

**Figure 1:**
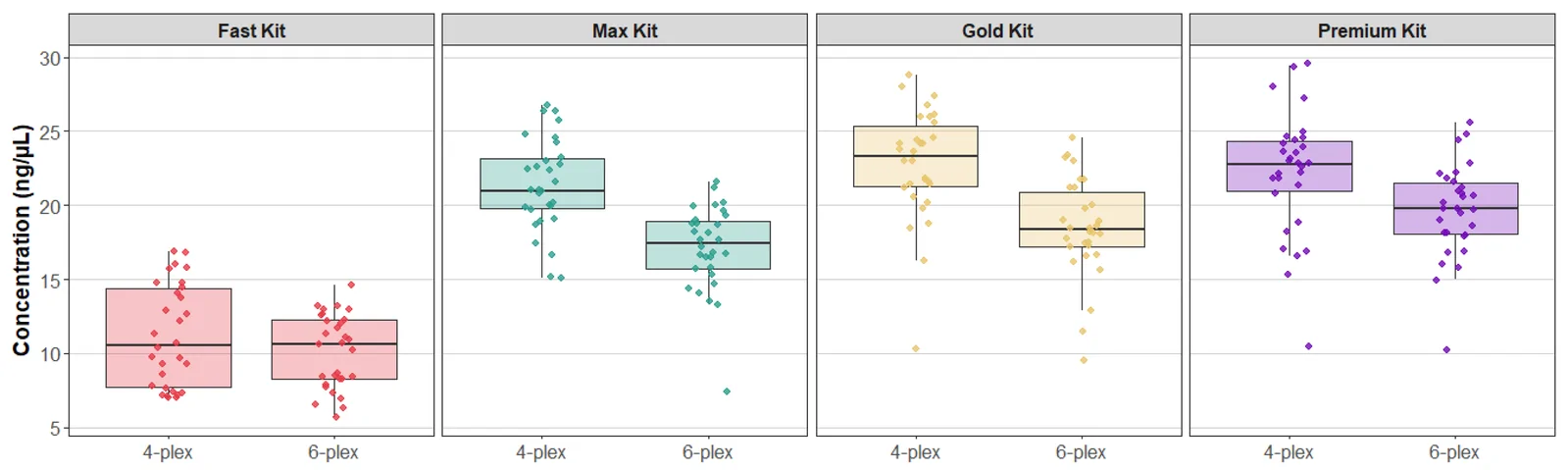
Boxplot of the 6-plex and 4-plex post-PCR concentrations (ng/µL) of the 50 ng cohort (DNA methylation standards and samples) across the four tested bisulfite conversion kits (Fast, Max, Gold, and Premium). Each dot represents a single QuBit measurement.

The PCR amplification of the 10 targets was carried out in two multiplexes with 4 and 6 targets [9]. Following the PCR, the amplified fragments were purified with the MinElute PCR Purification Kit (Qiagen) and quantified using the Qubit dsDNA High Sensitivity (HS) Assay Kit (Thermo Fisher Scientific). Results from the 50 ng cohort are shown in Figure 1.

The concentrations obtained with the 50 ng cohort and DNA methylation standards differed among the four bisulfite conversion kits. The Type III ANOVA test performed on the linear mixed models fitted on each multiplex, revealed significant effects of the bisulfite kit on the concentrations for both the 4-plex (F (3,102) = 75.2, *p* < 2.2 × 10^−16^, partial η^2^ = 0.688) and the 6-plex (F (3,102) = 65.9, *p* < 2.2 × 10^−16^, partial η^2^ = 0.660). BH-adjusted post-hoc pairwise comparisons showed that samples processed with the Fast kit yielded significantly lower concentrations than all the other kits (*p* < 0.0001), with mean values of 11.18 ng/µL (95% CI: 9.78–12.6, median 10.55 ng/µL) for the 4-plex and 10.15 ng/µL (95% CI: 9.01–11.3, median 10.65 ng/µL) for the 6-plex. The remaining three kits (Premium, Gold, and Max) did not significantly differ from each other for the 4-plex (*p* > 0.18). For the 6-plex, the samples processed with the Premium kit yielded the highest concentrations (mean 19.64 ng/µL, median 19.8 ng/µL), and the DNA amount was significantly higher compared to samples processed with the Max kit (*p* = 0.0014). Samples converted with the Gold kit did not differ significantly from the Premium or Max kits (all *p* > 0.086).

**Figure 2:**
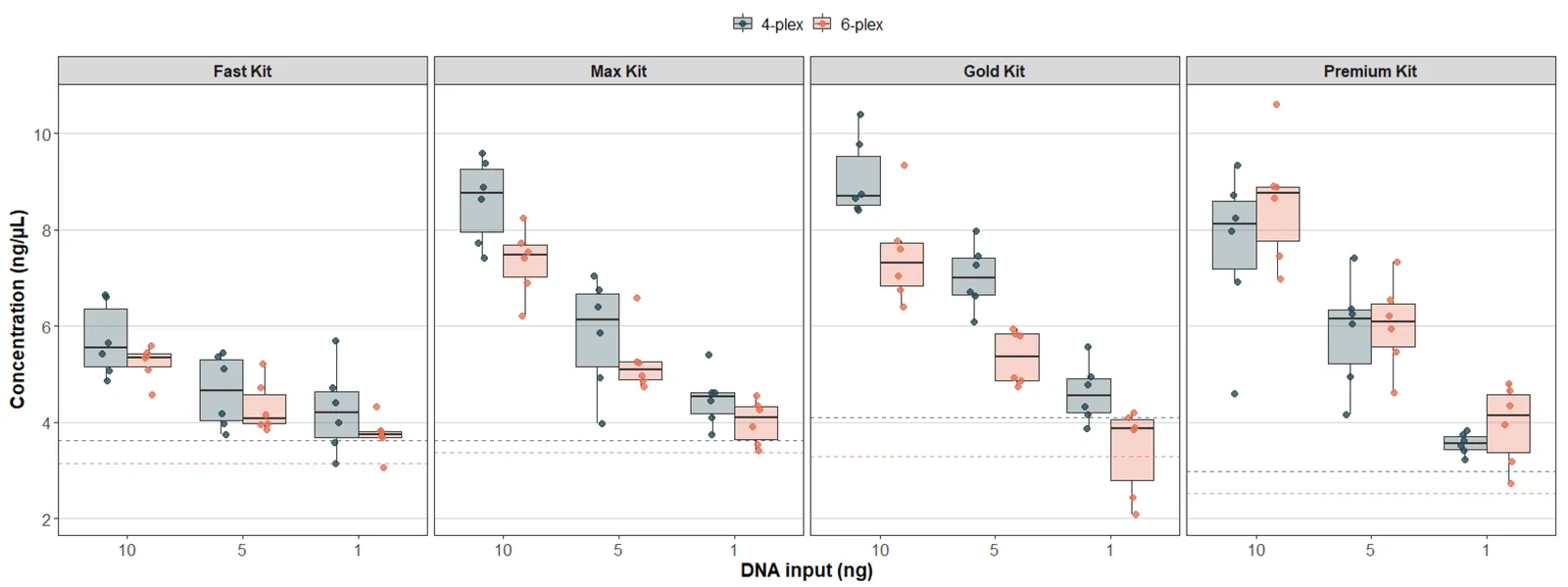
Boxplot of the concentrations of the two multiplexes (ng/µL) for the sensitivity cohort (DNA inputs: 10 ng, 5 ng, and 1 ng) across the tested bisulfite kits. The dashed lines represent the averaged QuBit concentrations of the 4-plex (blue) and 6-plex (orange) in the negative controls, that were used as a reference for background noise for each kit. Each dot represents a single QuBit measurement.

QuBit quantifications of the sensitivity cohort (samples with DNA inputs: 10 ng, 5 ng, and 1 ng) and negative controls were also assessed (Figure 2).

Wilcoxon signed-rank tests confirmed that all kits produced signals significantly above background (negative control signal) at every DNA input (all *p* < 0.05), except for the Fast kit treatment of the 4-plex (*p* = 0.109) and the Gold kit treatment of the 6-plex (*p* = 0.500) at 1 ng DNA input. The difference between the sample signal and background noise after treatment with the different bisulfite kits were compared using Kruskal-Wallis tests. No significant variance was found at 1 ng (4-plex: *p* = 0.644; 6-plex: *p* = 0.131), while significant kit effects were detected at 10 ng and 5 ng (all *p* < 0.021). As seen for the 50 ng cohort, Dunn post-hoc comparisons revealed that the concentrations after treatment with the Fast kit were significantly lower than the concentrations obtained with all other kits (all *p* values < 0.05) at 10 ng and 5 ng for both plexes. The Premium kit showed significantly higher concentrations for the 6-plex than all other kits (all BH-adjusted *p* < 0.036) at 10 ng and compared to the Max kit at 5 ng (BH-adjusted *p* = 0.030).

The significance of these results may be affected by the small number of samples per DNA input (Supplementary Table S2).

### 3.2. Bisulfite Conversion Efficiency

Bisulfite conversion efficiencies were compared across DNA inputs and bisulfite kits. As shown in Supplementary Figure S1, the bisulfite conversion efficiencies of the sensitivity cohort were comparable to those of the 50 ng cohort, which was confirmed by the Kruskal-Wallis and Dunn post-hoc comparison tests. The Fast and Premium kits showed no significant DNA input effect (*p* = 0.318 and *p* = 0.088, respectively) on the conversion efficiencies, while the Max and Gold kits presented only a small but significant higher bisulfite conversion efficiency in the 1 ng sensitivity samples compared to the 50 ng cohort (Max: BH-adjusted *p* = 0.031; Gold: BH-adjusted *p* = 0.012). These results confirmed that bisulfite conversion efficiency was largely stable across DNA inputs, with only a minor but positive difference at 1 ng for two of the four kits. Therefore, the sensitivity samples were included in the overall comparison (see below).

**Figure 3:**
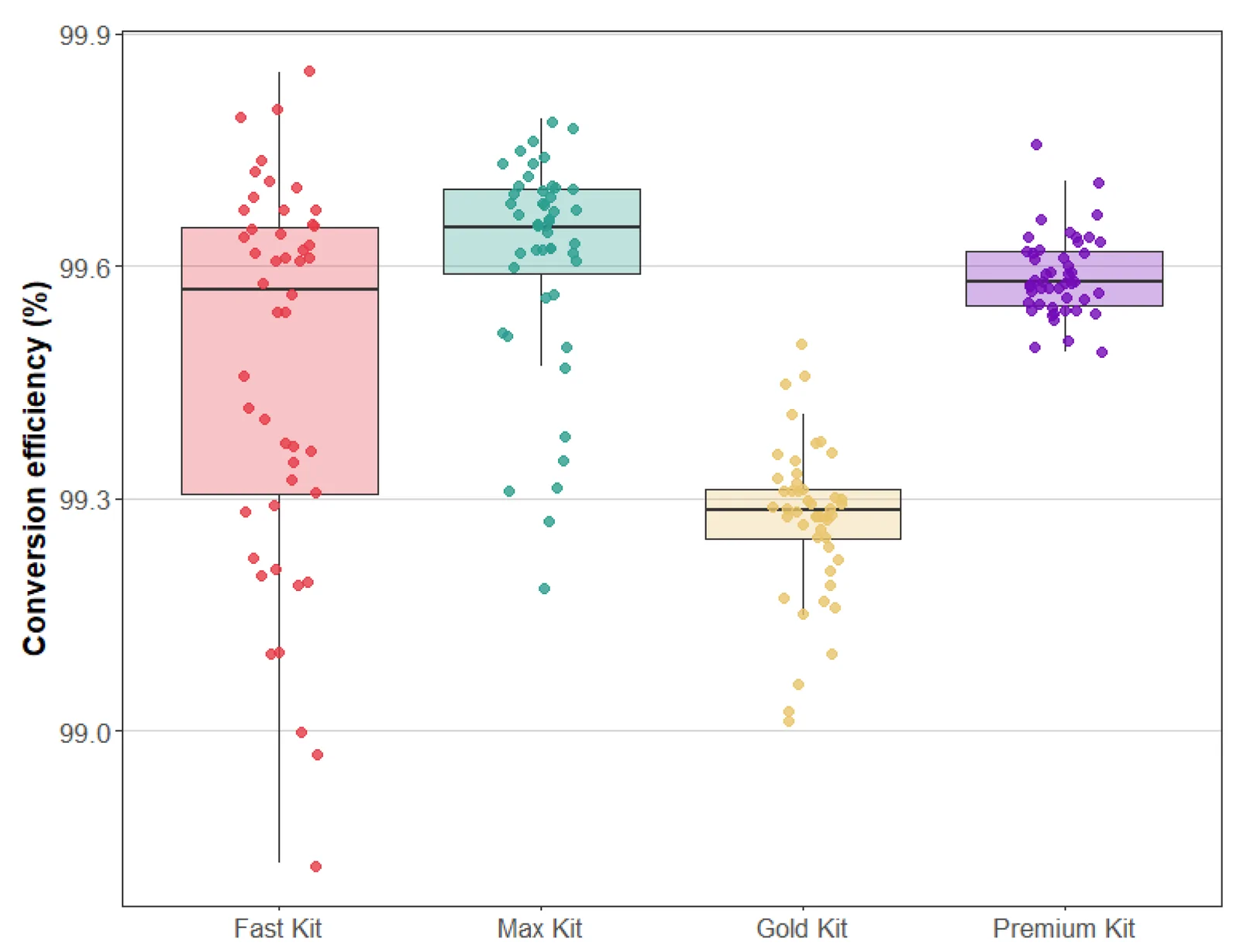
Boxplot of the bisulfite conversion efficiency (in %) of all the samples and DNA methylation standards across the four kits. Each dot represents a different sample or DNA methylation standard from the 50 ng or sensitivity cohorts.

The distribution of the average bisulfite conversion efficiency values is represented in Figure 3.

The Type III ANOVA test on the linear mixed model fitted on all the samples and DNA methylation standards found significant effects on the bisulfite conversion efficiency (F (3,165) = 55.05, *p* < 2.2 × 10^−16^, partial η^2^ = 0.500) of the bisulfite kits. The BH-adjusted pairwise comparisons subsequently revealed that the samples processed with Max and Premium kits yielded significantly higher bisulfite conversion efficiencies (means of 99.61% (95% CI: 99.57–99.66), and 99.59% (95% CI: 99.54–99.63), respectively) than samples processed with the Fast (99.47%, 95% CI: 99.43–99.52) and Gold kits (99.27%, 95% CI: 99.23–99.32, all pairwise *p* < 0.0002), without significantly differing from each other (*p* = 0.435). Additionally, the samples converted with the Fast kit had significantly higher bisulfite conversion efficiencies than samples treated with the Gold kit (*p* < 0.0001), but with higher variance, as shown in Figure 3.

### 3.3. Reproducibility

#### 3.3.1. Run-to-Run Reproducibility of Total and Normalized Read Depths

Read depths of the 10 amplicons were investigated across kits, DNA inputs, and sequencing runs.

In the 50 ng cohort, the Type III ANOVA test revealed a significant bisulfite kit effect on total read depth (F (3,102) = 12.73, *p* = 3.9 × 10^−7^, partial η^2^ = 0.27). Samples processed with the Max (mean 303,232 reads) and Premium (mean 283,568 reads) kits presented significantly higher total read depth than the Gold (mean 244,591 reads) and Fast (mean 227,940 reads) kits (all BH-adjusted *p* < 0.001), with no significant difference between Max and Premium (*p* = 0.186) or between Gold and Fast (*p* = 0.228). Nevertheless, the read depths of most fragments were above the usually applied threshold of 1000 reads, with three exceptions in samples processed with the Fast kit (MIR29B2CHG in Sample 1–10 ng and 1 ng DNA input, and ARHGAP22 in Sample 5–1 ng DNA input). Normalized read depth uniformity across the 10 amplicons also differed significantly by kit (F (3,102) = 58.8, *p* < 2.2 × 10^−16^, partial η^2^ = 0.63): the Max kit showed the most uniform distribution (mean SD 0.054), significantly better than all other kits (all BH-adjusted *p* < 0.0001), while the Fast kit showed the greatest variability (mean SD 0.072, all BH-adjusted *p* < 0.001) compared to the other kits.

At lower DNA inputs, total read depth did not differ significantly between kits (Kruskal-Wallis, all *p* > 0.07), likely reflecting the limited sample size at each input level (Supplementary Table S2). In contrast, normalized read depth uniformity showed significant kit differences at every input level (all *p* < 0.035), consistently driven by the Fast kit’s poorer uniformity relative to the other three kits (all BH-adjusted *p* > 0.055 among Max, Gold, and Premium). Within each kit, total read depth decreased significantly at 1 ng input relative to higher inputs (Fast: F (3,18) = 6.77, *p* = 0.003, partial η^2^ = 0.53; Max: F (3,20) = 4.33, *p* = 0.017, partial η^2^ = 0.39; Gold: F (3,18) = 9.83, *p* < 0.001, partial η^2^ = 0.62; Premium: F (3,20) = 3.69, *p* = 0.029, partial η^2^ = 0.36).

More detailed information on total and normalized read depth distribution is presented in Supplementary File S1.

#### 3.3.2. Run-to-Run Reproducibility of β-Values

**Figure 4:**
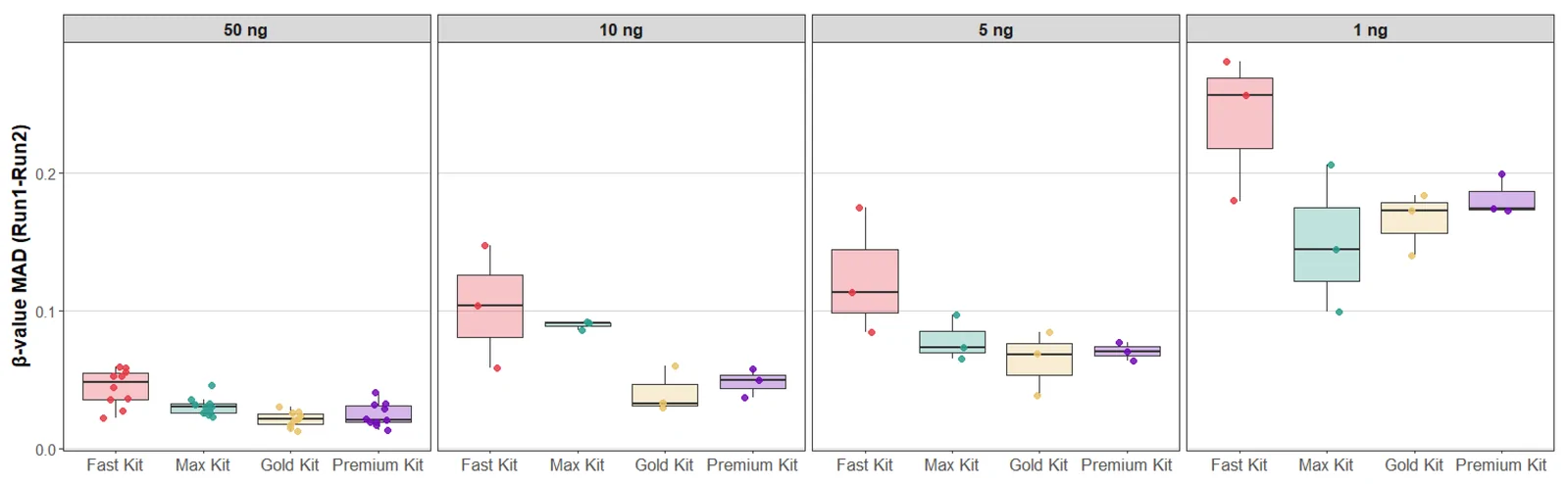
Boxplots of the mean absolute difference (MAD) in β-values between sequencing runs, calculated across the 11 CpGs of each sample (50 ng and sensitivity cohort).

Samples from the 50 ng cohort and from the sensitivity cohort were investigated in their β-value reproducibility across runs. Mean absolute differences between the β-values are shown in Figure 4.

The Type III ANOVA test on the linear mixed model fitted on the MADs found a significant bisulfite kit effect on the β-value differences between runs (F (3,54) = 14.38, *p* < 0.00001, partial η^2^ = 0.444; 95% CI: Fast 0.120 [0.017–0.223], Max 0.089 [−0.014–0.192], Gold 0.077 [−0.026–0.180], Premium 0.083 [−0.020–0.186]). The BH-adjusted pairwise comparison found that samples processed with the Fast kit had significantly worse β-value reproducibility than all other kits (all BH-adjusted *p* < 0.0001), whereas no significant differences were revealed among the other kits (all BH-adjusted *p* > 0.12). The Gold and Premium kits maintained the lowest MAD values at 10 and 5 ng, while the Max kit showed the lowest MAD at 1 ng. MAD increased substantially as DNA input decreased across all kits, with values rising from 0.021 to 0.045 at 50 ng to 0.150–0.239 at 1 ng, indicating that all kits became considerably less reproducible at lower DNA inputs.

### 3.4. β-Value Specificity

#### 3.4.1. β-Values in the DNA Methylation Standards

**Figure 5:**
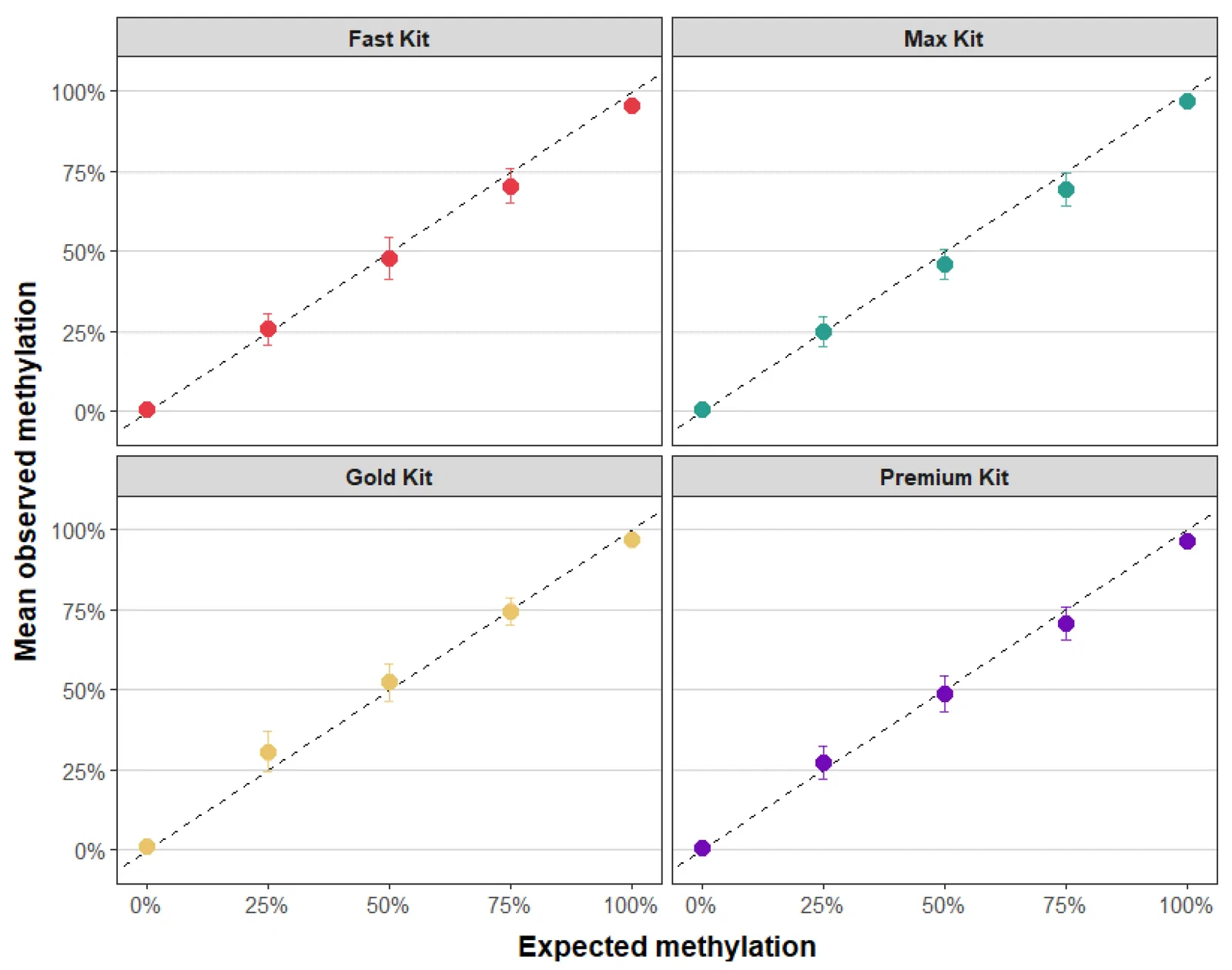
Expected methylation values (0%, 25%, 50%, 75%, and 100%) plotted against the observed β-values of the 11 CpGs. The results were averaged between runs for the four bisulfite conversion kits (Fast, Max, Gold, and Premium). Error bars represent ±1 SD across CpGs. Dots lying on the dashed line present a perfect estimation of the mean methylation percentage across the 11 CpG sites.

The β-values of the five DNA methylation standards (0%, 25%, 50%, 75%, and 100%) across the 11 CpGs of the original age prediction model [9,29] were averaged, and their absolute difference from the expected methylation percentages was calculated. The mean absolute error (MAE) was determined, averaging across duplicate runs (Figure 5).

The Type III ANOVA test on the linear mixed model revealed no significant effect of bisulfite kit on the absolute error (F (3,32) = 0.19, *p* = 0.904, partial η^2^ = 0.02), while the expected methylation level significantly affected the accuracy (F (4,32) = 3.56, *p* = 0.016, partial η^2^ = 0.31). The BH-adjusted pairwise comparisons confirmed no statistical difference between the kits (all BH-adjusted *p* > 0.96). The overall mean absolute errors ranged from 2.42 (Premium kit) to 2.99 (Max kit) percentage points. Slightly worse performance was observed in the 75% and 100% DNA methylation standards.

#### 3.4.2. β-Values in the Sensitivity Cohort

**Figure 6:**
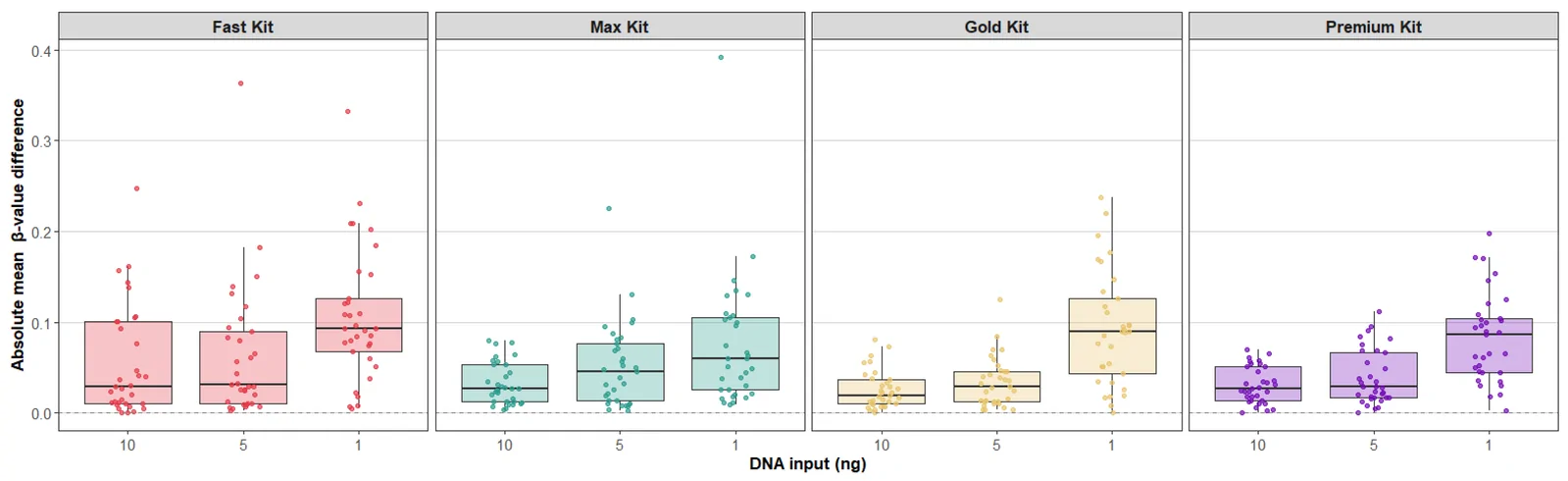
Boxplots of the absolute difference in mean β-values between the methylation levels of the 11 CpGs in the sensitivity samples and the corresponding ones from the 50 ng cohort. Dots lying on the bottom dashed line are CpGs whose mean beta value was equal to the mean beta value in the 50 ng reference sample.

To assess how the reduced DNA input affected methylation specificity, the β-values of the sensitivity samples were compared to the β-values of the corresponding sample in the 50 ng cohort (Figure 6).

The Kruskal-Wallis tests performed separately at each DNA input level (10 ng, 5 ng, and 1 ng) revealed no significant differences in β-values among the four kits (all *p* > 0.2). Overall, as can be observed in Supplementary Table S3 and Figure 6, the mean absolute difference in β-values increased with lower DNA input. The highest β-value differences were found in the 1 ng samples, while similar differences were present at 10 ng and 5 ng DNA input. The samples treated with the Gold kit yielded the most accurate β-values at 10 and 5 ng DNA inputs (mean |Δβ| = 0.026 and 0.034, respectively), whilst the Max kit was the most accurate at 1 ng input (mean |Δβ| = 0.075). Samples processed with the Fast kit consistently showed the largest deviations from the 50 ng cohort at all input levels, although these differences did not reach statistical significance. Since these values are not fully independent within a donor, the Kruskal-Wallis test may not appropriately capture the true uncertainty around these estimates, regardless of statistical significance, and these results should be interpreted with caution.

### 3.5. Age Prediction

Age prediction, a practical application of DNA methylation analyses, was used as an additional comparison metric for the four bisulfite conversion kits. Age predictions were performed with the 11-CpG model [9] and the SVMp-34CpG model [28]. Predictions across duplicate runs were averaged, and the MAEs between predicted and chronological age were calculated per kit and DNA input (Table 2).

Visual representation of the distribution of the absolute errors in the predictions and their resulting MAE is shown in Supplementary Figure S2. The Type III ANOVA tests on linear mixed models revealed no significant effect of the bisulfite kit on the prediction error in the 50 ng cohort for either model (11-CpG: F (3,27) = 1.30, *p* = 0.294, partial η^2^ = 0.13; SVMp-34CpG: F (3,27) = 0.45, *p* = 0.717, partial η^2^ = 0.05). At lower DNA inputs, no significant differences were found between bisulfite kits using the Kruskal-Wallis tests (all *p* > 0.05). The significance of the sensitivity cohort results may be affected by the small number of samples per DNA input (Supplementary Table S2).

The 11-CpG and SVMp-34CpG models were compared to establish the best performing model by using a linear mixed model with model type and DNA input as fixed effects, and sample and kit as random effects. The SVMp-34CpG model showed a lower error than the 11-CpG model in the 50 ng cohort (MAE 1.89 and 2.37 years, respectively), but it did not reach statistical significance (F (1,69) = 2.70, *p* = 0.105, partial η^2^ = 0.038). In contrast, slightly higher MAEs were observed for the SVMp-34CpG model than for the 11-CpG model at 5 and 1 ng DNA input. However, these differences were also not significant (all BH-adjusted *p* > 0.38).

To determine the DNA input threshold at which the prediction accuracy significantly decreased, pairwise comparisons between input levels were made. Independently of the model, predictions at 1 ng were significantly worse than at all other input (1 ng vs. 5 ng: 6.53 years, 95% CI 3.20–9.86; 1 ng vs. 10 ng: 7.39, 95% CI 4.06–10.72; 1 ng vs. 50 ng: 8.59, 95% CI 5.81–11.38; all BH-adjusted *p* < 0.0001), while no significant differences were observed between 50 ng, 10 ng and 5 ng DNA inputs (all 95% CIs crossed zero; all BH-adjusted *p* > 0.05). Thus, regardless of the bisulfite conversion kit or the prediction model used, 5 ng DNA input appeared to be the minimum recommended input.

## 4. Discussion

In this study, four bisulfite conversion kits—the Premium Bisulfite Kit, the SuperMethyl™ Fast Bisulfite Conversion Kit, the SuperMethyl™ Max Bisulfite Conversion Kit, and the EZ DNA Methylation-Gold™ Kit—were tested and compared by including them in an assay for forensic age prediction that relies on DNA methylation markers [9]. A total of five DNA methylation standards (0%, 25%, 50%, 75%, 100%), 10 blood samples with 50 ng DNA input (50 ng cohort), and nine sample-input combinations—three donors, each at 10 ng, 5 ng, and 1 ng DNA input (sensitivity cohort)—were processed in duplicates with each bisulfite conversion kit, and the bisulfite treated DNA was subsequently amplified by PCR and sequenced.

Across all the metrics used for comparison, no single kit emerged as having superior performance. Post-PCR concentrations were comparable across the Gold, Max, and Premium kits, and the samples presented overall high DNA concentrations when processed with these kits (Figure 1). Significantly lower amounts of PCR products were obtained for samples treated with the Fast kit. At 1–10 ng DNA inputs, the highest DNA concentrations were obtained with the Premium and Max kits, highlighting their better suitability for samples with low amounts of DNA (Figure 2).

The significant better performance of the Max and Premium kits was apparent by the bisulfite conversion efficiencies (Figure 3), where the Max kit yielded the highest values (mean: 99.6%). Nevertheless, all kits achieved high bisulfite conversion efficiencies (>99%), with the differences lying within a 1% interval. Incomplete bisulfite conversion is expected to introduce a bias in quantifying DNA methylation. However, at the conversion efficiencies observed in this study, this bias would affect the β-values by less than one percentage point. Consistent with this, the choice of bisulfite kit had no significant effect on the β-value specificity or age prediction accuracy, suggesting that the observed differences in conversion efficiency, while statistically significant, do not translate into meaningful differences in methylation estimation or age prediction. Additionally, the Fast, Gold and Premium kits met the expected bisulfite conversion efficiencies stated by their manufacturer (>99.5%, >99%, and >99.5% respectively), whereas the Max kit fell short of its stated specification (>99.8%).

Reproducibility of total read depth, normalized read depth, and β-values between experiments was comparable for samples processed with the Gold, Premium, and Max kits, whereas the Fast kit presented significantly lower reproducibility between runs, across all DNA inputs.

As shown in previous studies, bisulfite conversion kits can differ substantially despite similar chemistries [17,19,20,21,23]. The Fast kit’s brief bisulfite incubation time may be the reason for the lower performance, as rapid but harsher bisulfite treatments lead to poorer DNA yields and higher fragmentation rates [16,19]. For laboratories prioritizing high DNA recovery after bisulfite treatment and uniform sequencing depth between runs, this study indicated that the Fast kit was the least suitable. In contrast, the Max and Premium kits seemed to show more robust performances across the investigated technical metrics, particularly at lower DNA inputs.

The choice of bisulfite conversion kits had no statistically significant effect on the two most important metrics: β-value specificity and age prediction accuracy. However, the statistical power might have been limited by the number of samples used for these statistical analyses. Descriptively, the estimated β-values of the DNA methylation standards were within a narrow error margin (MAE 2.4–3.0 percentage points) for all four kits, and this was maintained at lower DNA inputs (mean |Δβ| < 0.1). The samples processed with the Gold kit yielded the most accurate β-values at 10 ng and 5 ng DNA input, while samples with 1 ng DNA input had the most accurate β-values when processed with the Max kit.

Age prediction performance was statistically indistinguishable among the four bisulfite kits, both with the 11-CpG model [9] and the SVMp-34CpG model [28]. Indeed, the four kits had similar performance in the 50 ng cohort, suggesting that, despite the differences in technical metrics, all bisulfite kits yielded similar results in samples with optimal DNA inputs (Table 2; Supplementary Figure S2). At lower DNA inputs, however, the kits’ performance in prediction accuracy differed. Descriptively, samples processed with the Gold, Premium and Max kits yielded higher age prediction accuracies, particularly at 5 ng and 1 ng DNA input. These differences did not reach statistical significance, likely due to the limited number of samples analyzed, and should be interpreted as trends rather than confirmed effects. Among the two prediction models, the SVMp-34CpG presented lower MAEs at the optimal DNA input but was less accurate in predicting the age of the samples in the sensitivity cohort. This suggests that the higher number of CpGs used to build the model negatively affected the age predictions when the determination of the methylation levels was more uncertain.

The 1 ng samples presented lower performance and uniformity across all technical metrics (Figure 2, Figure 4 and Figure 6, Supplementary File S1) and significantly lower age prediction accuracy (Table 2 and Supplementary Figure S2), whereas the samples with 50 ng, 10 ng and 5 ng DNA input were statistically indistinguishable from each other regardless of the bisulfite kit or model used for age prediction. This showed that 5 ng DNA should be the minimum recommended input for the age prediction assay used here. The threshold represents valuable information for forensic casework, where the DNA is often limited and degraded [37], and is in accordance with the conclusion made by the original developers of the assay [29]. This threshold is, however, based on a limited number of samples and requires validation in larger cohorts as well as real forensic casework samples.

For forensic casework, reproducibility and robustness at low DNA inputs are the main deciding factors in selecting the appropriate bisulfite kit. This study suggests that the Max or Premium kits are preferable for forensic analyses due to their better performance within these metrics, whereas the Fast kit may be less suitable. It should be noted, however, that no statistically significant differences were found between kits for age prediction accuracy, and this suggestion should therefore not be interpreted as evidence that one kit yields more accurate age predictions than another. In clinical settings, where sample quantity is rarely a constraint or a highly precise estimation of methylation values might not be required, the choice of kit may be different. The Fast kit may represent a perfectly reasonable choice given its lower cost and shorter incubation step (Table 1). The Premium and Gold kits would be intermediate choices based on cost and time factors, as they both present shorter hands-on time, but longer incubation steps and higher costs than the Fast kit. We therefore caution against interpreting the results of this study as a recommendation against the Fast kit, as kit selection should be guided by the specific demands of the intended application.

## Figures and Tables

**Table 1:** Laboratory protocol differences in the four tested bisulfite conversion kits.

Kit	Thermocycler Incubations ^1^		Methodology	Optimal DNA Input (ng) ^2^	Elution Volume (µL)	Hands-On Time ^3^	Price ^4^
Max	20 min at 42 °C	90 min at 55 °C <5 min at 4 °C	Beads	0.1–2000	25	***	***
Premium	8 min at 98 °C 60 min at 54 °C	/	Columns	200–500	10	*	**
Gold	10 min at 98 °C, 150 min at 64 °C	/	Columns	200–500	10	*	**
Fast	7 min at 98 °C	/	Beads	100–1000	25	***	*

^1^ Number of incubation steps required in each protocol with corresponding incubation time and temperatures. ^2^ Recommended in the manufacturer’s protocol. ^3^ Evaluated as hands-on time expenditure. A higher number of stars indicates longer hands-on time. ^4^ Evaluated as price per sample. A higher number of stars indicates a higher price.

**Table 2:** Mean absolute errors (MAEs) of the age predictions obtained with the 11-CpG and the SVMp-34CpG models across the different kits and DNA inputs.

DNA Input (ng)	Fast ^1^		Max		Gold		Premium	
	11-CpG ^2^	SVMp-34CpG	11-CpG	SVMp-34CpG	11-CpG	SVMp-34CpG	11-CpG	SVMp-34CpG
50	2.77	2.17	1.86	2.00	2.69	1.69	2.17	1.71
10	3.24	5.04	4.57	3.35	2.07	3.86	3.63	0.90
5	5.09	9.72	2.15	3.06	3.11	1.57	4.39	4.48
1	15.56	23.24	9.66	5.67	9.66	9.52	4.93	7.56

^1^ Bisulfite conversion kit. ^2^ MAE (years) of the specified age prediction model.

## Data Availability

The data presented in this study are available in the article and Supplementary Material.

## Supplementary Materials

The following supporting information can be downloaded at: https://www.mdpi.com/article/10.3390/genes17091100/s1, Figure S1: Boxplots of the bisulfite conversion efficiency (in %) at different DNA inputs (50 ng, 10 ng, 5 ng, and 1 ng); Figure S2: Boxplots displaying the distribution of the absolute errors (in years) obtained from the age predictions using the 11-CpG and SVMp-34CpG models across different the four bisulfite conversion kits (Fast, Max, Gold, Premium) and DNA inputs (50 ng, 10 ng, 5 ng, and 1 ng); Table S1: DNA input and concentration of the samples and DNA methylation standards; Table S2: Summary of statistical tests performed for each section of the data analysis and the number of samples used; Table S3: Averages of the absolute mean beta differences calculated between the sensitivity samples and the 50 ng reference samples; File S1: Results of the analysis of the run-to-run reproducibility of total and normalized read depths.
