# Supplementary material for: Comparison of Four Bisulfite Conversion Kits and Their Effects on DNA Methylation Detection and Age Prediction
Source: Genes (Basel). 2026 Sep 11;17(9):1100. doi: 10.3390/genes17091100 (PMC13606544; doi:10.3390/genes17091100)
Supplement: Supplementary file 1 [file genes-17-01100-s001.zip › Supplementary Figures.pdf]

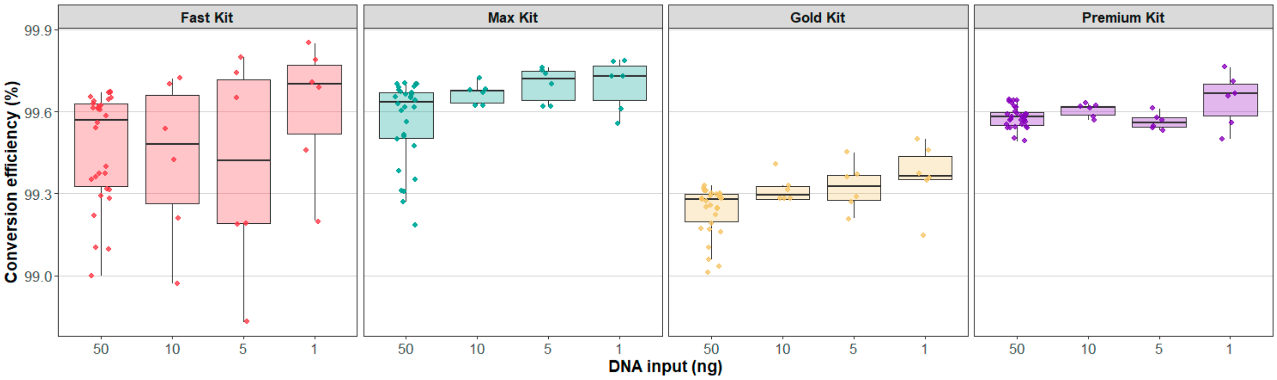

Supplementary Figure S1: Boxplots of the bisulfite conversion efficiency (in %) at different DNA inputs (50 ng, 10 ng, 5 ng, and 1 ng) across the four bisulfite conversion kits. Each dot represents a sample or methylation standard.

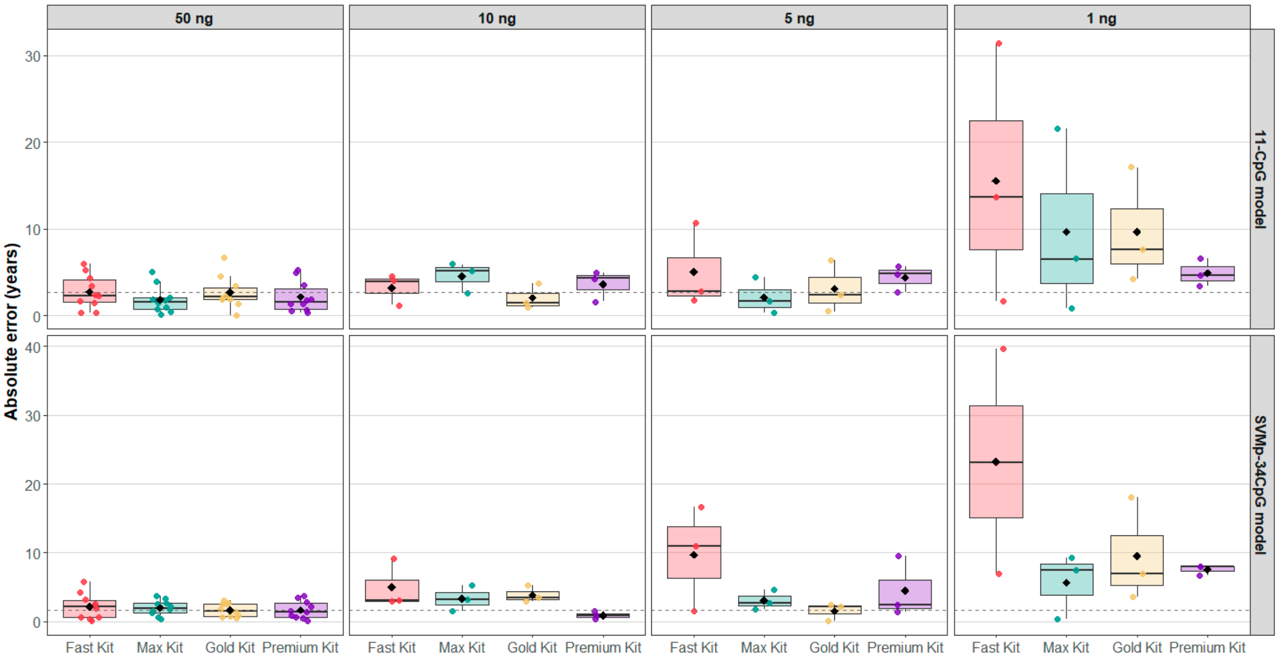

Supplementary Figure S2: Boxplots displaying the distribution of the absolute errors (in years) obtained from the age predictions using the 11-CpG and SVMp-34CpG models across the four bisulfite conversion kits and DNA inputs (50 ng, 10 ng, 5 ng, and 1 ng). The black dots represent the Mean Absolute Error (MAE). The dashed lines represent the MAE of the models from their respective validation datasets (2.7 years for the 11-CpG model and 1.7 years for the SVMp-34CpG model).
