# Supplementary material for: Comparison of Four Bisulfite Conversion Kits and Their Effects on DNA Methylation Detection and Age Prediction
Source: Genes (Basel). 2026 Sep 11;17(9):1100. doi: 10.3390/genes17091100 (PMC13606544; doi:10.3390/genes17091100)
Supplement: Supplementary file 1 [file genes-17-01100-s001.zip › Supplementary File S1.pdf]

Supplementary File S1 - Results of the analysis of the Run-to-run reproducibility of total and normalized read depths

Total read depths were investigated across kits, DNA inputs, and sequencing runs. The Type III ANOVA test on the linear mixed model output revealed significant bisulfite kit effects on the total read depth in the 50 ng cohort ($F(3,102) = 12.73$, $p = 3.9 \times 10^{-7}$, partial $\eta^2 = 0.27$). The pairwise bisulfite kit comparisons showed that samples processed with the Max (mean total read depth 303,232, 95% CI: 283,914–322,551) and Premium (283,568, 95% CI: 264,249–302,887) kits presented significantly higher read depths than samples processed with the Gold (244,591, 95% CI: 225,272–263,909) and Fast kits (227,940, 95% CI: 208,621–247,259) (Max–Gold: 58,642, 95% CI 21,722–95,562; Max–Fast: 75,292, 95% CI 38,372–112,212; Premium–Fast: 55,628, 95% CI 18,708–92,548; all BH-adjusted $p < 0.001$). No significant differences were observed between the Max and Premium ($p = 0.186$) or the Gold and Fast kits ($p = 0.228$). At lower DNA inputs (10-1 ng), no significant differences in total read depths were detected between kits (Kruskal-Wallis, all $p > 0.07$). This may be due to the limited number of samples per kit that reduced the statistical power (Supplementary Table S2). The highest mean total read depth was obtained with the Premium kit at all DNA inputs (327,876 at 10 ng, 329,447 at 5 ng, and 187,234 at 1 ng), as shown in Image 1.

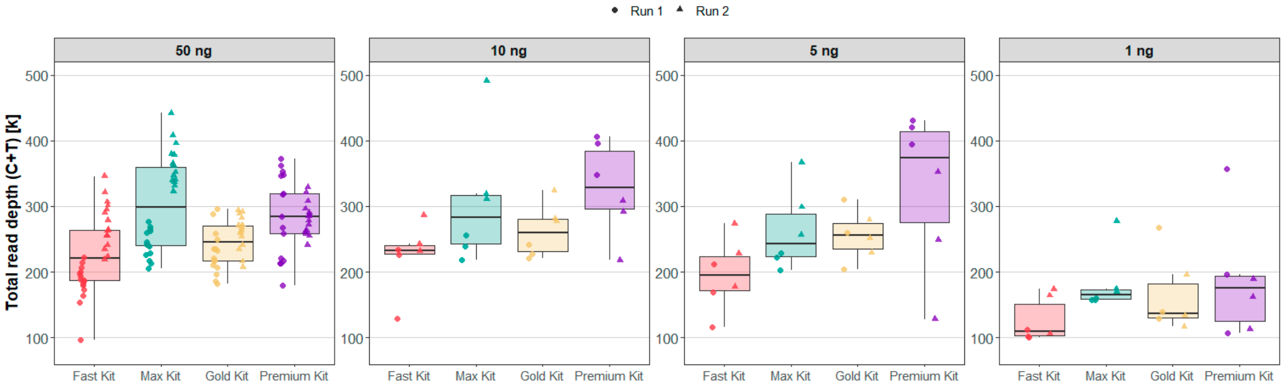

Image 1: Distribution of total read depth (C+T) calculated across the 10 amplicons at different DNA inputs (50 ng, 10 ng, 5 ng, and 1 ng).

The sensitivity samples and their corresponding 50 ng samples were compared within each kit using linear mixed models with sample as a random effect, to assess the impact of the DNA input on the total read depths. The Type III ANOVA test on the linear mixed models showed a significant DNA input effect on all four kits (Fast: $F(3,18) = 6.77$, $p = 0.003$, partial $\eta^2 = 0.53$; Max: $F(3,20) = 4.33$, $p = 0.017$, partial $\eta^2 = 0.39$; Gold: $F(3,18) = 9.83$, $p < 0.001$, partial $\eta^2 = 0.62$; Premium: $F(3,20) = 3.69$, $p = 0.029$, partial $\eta^2 = 0.36$). For all kits, read depths at 1 ng input were significantly lower than at 50 ng and 10 ng input (all BH-adjusted $p < 0.05$). For the Fast, Gold, and Premium kits, read depths at 1 ng input was also significantly lower than at 5 ng input (all $p \leq 0.036$), while this difference was not significant for the Max kit ($p = 0.147$). The significance of these results may be affected by the small number of samples per DNA input (Supplementary Table S2).

The normalized read depth was calculated as the ratio of reads (C+T) in each amplicon divided by the total read depth of the sample or DNA methylation standard. The distributions of the normalized read depth in the 50 ng cohort are shown in Image 2.

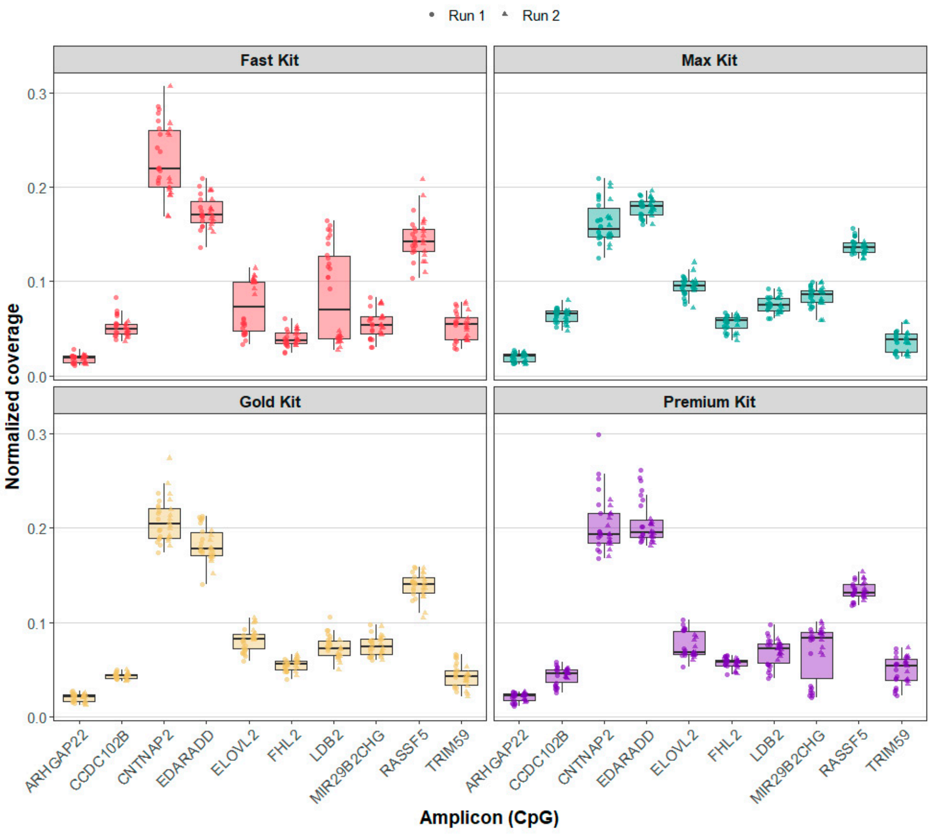

Image 2: Boxplots of the normalized read depths of the 10 amplicons in the 50 ng cohort across bisulfite conversion kits and sequencing runs.

The standard deviation (SD) of the normalized read depths of the 10 amplicons was calculated. The Type III ANOVA test on the linear mixed model confirmed that the bisulfite kit significantly influenced the SD of normalized read depths ($F(3,102) = 58.8$, $p < 2.2 \times 10^{-16}$, partial $\eta^2 = 0.63$), while the BH-adjusted pairwise comparisons revealed that samples processed with the Max kit had significantly more uniform distributions of normalized read depth (mean SD 0.054) than all the other kits (all BH-adjusted $p < 0.0001$), whereas the Fast kit (mean SD 0.072) had significantly more variation than the others (all BH-adjusted $p < 0.001$). The Gold kit showed similar performance to the Premium kit, with only a slightly more uniform distribution (Gold mean SD 0.064, Premium mean SD 0.067, $p = 0.043$).
The SD of the normalized read depth in the sensitivity cohort and their corresponding 50 ng sample is shown in Image 3.

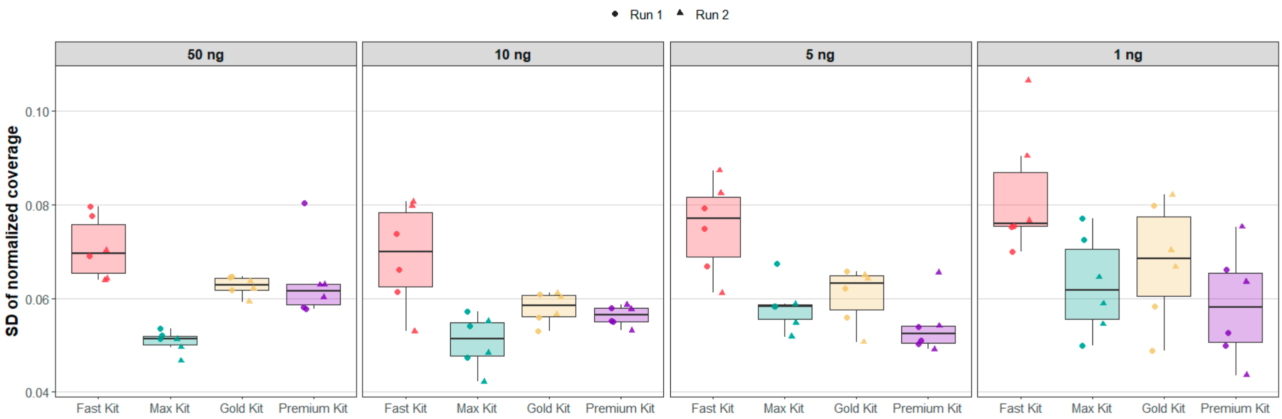

Image 3: Boxplots of the SD of the normalized read depth for the sensitivity cohort (DNA inputs: 10 ng, 5 ng, and 1 ng) and the corresponding 50 ng sample across different bisulfite conversion kits. The round and triangular shapes distinguish the two sequencing runs.

At low DNA inputs (10 ng, 5 ng, and 1 ng), the Kruskal-Wallis tests detected significant kit differences at all input levels (all $p < 0.035$). Dunn post-hoc comparisons (BH-adjusted) confirmed that the samples converted with the Fast kit had the least uniform read depth whereas there were no significant differences between the Premium, Gold and Max kits at any DNA input (all BH-adjusted $p > 0.055$). The significance of these results may be affected by the small number of samples per DNA input (Supplementary Table S2).
